# The Effect of ADHD Stimulant Treatment on Weight Categories in Children and Adolescents

**DOI:** 10.3390/jcm15010044

**Published:** 2025-12-21

**Authors:** Shlomit Yaron, Ronen Arbel, Talish Razi, Dan Nemet

**Affiliations:** 1 Schneider Children’s Medical Center of Israel, P.O. Box 559, Petach Tikvah 4920235, Israel; yshlomit@clalit.org.il; 2 Clalit Health Services, Community Medical Services Division, 101 Arlozorov Street, Tel Aviv 6209804, Israel; 3 Maximizing Health Outcomes Lab, Sapir College, Sderot 7915600, Israel; 4 Child Health and Sports Center, Department of Pediatrics, Meir Medical Center, 59 Tchernichovski Street, Kfar-Saba 44281, Israel; 5 School of Medicine, Gray Faculty of Medical and Health Sciences, Tel Aviv University, Tel Aviv 69978, Israel

**Keywords:** attention-deficit hyperactivity disorder, medications, weight, growth

## Abstract

**Objective:** Pediatric overweight and obesity represent a growing public health concern with significant long-term implications. In children diagnosed with attention-deficit/hyperactivity disorder (ADHD), stimulant medications may alter appetite, potentially impacting body weight and growth patterns. However, real-world data on the effect of these treatments on body mass index (BMI) classification remains scarce. We aimed to evaluate the effect of ADHD stimulant therapy on transitions in the BMI categories among children. **Study Design:** We conducted a large-scale observational cohort study assessing longitudinal changes in BMI classification following the initiation of stimulant treatment, utilizing data from Clalit Health Services, Israel’s largest healthcare provider. BMI was categorized into four groups: normal weight, overweight, obesity, and severe obesity. Subgroup analysis was performed by sex and age groups: <7 years; >7 <13 years and >13 <18 years. **Results:** At baseline, 26,930 children met the study inclusion criteria. 12,448 (46%) were classified as overweight or obese. Most children with normal weight at baseline maintained their BMI classification (90%). 48% of children with overweight, 42% with obesity, and 29% with severe obesity transitioned to a lower BMI category. 39% of children with underweight transitioned to normal weight. Similar patterns in BMI category transitions were observed between sexes. Transition to a lower BMI category was more prevalent in the younger age group. **Conclusions:** Stimulant therapy for ADHD is associated with significant shifts in BMI classification among pediatric patients. While many children, especially younger with higher baseline BMI, experienced improvements in weight status, a notable minority exhibited weight gain. These findings underscore the importance of routine BMI monitoring and weight management strategies during ADHD treatment.

## 1. Introduction

Attention-deficit/hyperactivity disorder (ADHD) is one of the most prevalent neurodevelopmental disorders in childhood, commonly managed with stimulant medications such as methylphenidate or amphetamines [1]. These medications are known to suppress appetite and may lead to reduced caloric intake, particularly during the initial stages of treatment. Consequently, weight loss is a documented side effect of stimulant therapy in children [2,3].

Pediatric obesity is another growing health concern worldwide. Despite intense efforts to maintain proper body weight, prevent weight gain, or promote weight loss, the incidence of obesity and its complications among children and adolescents is growing to epidemic proportions. Pediatric obesity is linked to an increased risk of dyslipidemia, insulin resistance, Type II diabetes, hypertension, and the development of coronary heart disease at a later stage in life, but also to significant health and psychological complications during childhood [4].

While stimulants often result in weight reduction, their impact on children and adolescents with overweight or obesity remains unclear. Evidence regarding sustained weight loss and changes in BMI classification in this population is limited and inconsistent. Schwartz et al. demonstrated that ADHD in childhood, not treated with stimulants, was associated with higher childhood BMI. In contrast, they showed that children with ADHD treated with stimulants had a slower early BMI growth but a rebound later in adolescence to levels that are above those of children without a history of ADHD or stimulant use [5]. On the other hand, Lewis et al. suggested that children who begin stimulant treatment for ADHD in late childhood and particularly early adolescence are at an increased risk for weight gain [6].

Stimulant medications exert their therapeutic effects primarily by increasing synaptic concentrations of dopamine and norepinephrine in the central nervous system. These neurotransmitters play a key role not only in attention regulation but also in appetite control, reward processing, and energy balance. Preclinical animal studies have demonstrated reduced food intake, altered hypothalamic signaling, and changes in reward-related feeding behavior following stimulant exposure, providing biological plausibility for stimulant-associated weight changes observed in clinical practice. However, compensatory behavioral and metabolic adaptations over time may attenuate these effects, particularly among children with higher baseline BMI, contributing to heterogeneous weight trajectories [7].

In light of the rising prevalence of pediatric obesity, the increased risk for obesity in children with ADHD [8], and the widespread use of stimulant medications, it is essential to evaluate their real-world impact on weight trajectories. This study aims to assess changes in BMI classification following the initiation of ADHD stimulant therapy, with particular attention to children and adolescents who are overweight or obese at baseline.

## 2. Materials and Methods

### 2.1. Study Design and Data Source

This retrospective, observational cohort study was conducted using data from Clalit Health Services, Israel’s largest health maintenance organization, which covers over 50% of the national population (4.9 million). The study utilized anonymized electronic medical records (EMRs) from Clalit’s central data repository, which includes comprehensive demographic, clinical, diagnostic, and prescription data.

Children were included if they were dispensed stimulant medications for at least 90 days within a year. This threshold was chosen to account for school holidays and vacation periods and to increase the likelihood of sustained exposure rather than short-term or trial use.

Height and weight measurements were obtained during routine clinical visits, using calibrated height stadiometers and weight scales, and recorded by healthcare professionals in the electronic medical record; self-reported measurements were not included.

Age groups (<7, 7–12, ≥13 years) were selected to reflect key developmental and educational stages corresponding to preschool age, elementary school years, and adolescence, which may influence eating behaviors, growth patterns, and response to pharmacologic treatment.

### 2.2. Study Population

Children and adolescents aged 5 to 18 years who initiated treatment with stimulant medications for ADHD between 1 January 2009, and 31 December 2023, were eligible for inclusion. Participants were divided into three age groups at the time of treatment initiation: Preschool (<7 years), Elementary school (>7 <13 years), and High school (>13 <18 years).

Only patients with recorded BMI measurements both before and after treatment initiation (within 12 months) were included.

Patients were included if they were dispensed stimulant medications for at least 90 days within a year, to account for school holidays and vacations. Patients who had undergone bariatric surgery or were prescribed GLP-1 receptor agonists were excluded, as these interventions may affect weight.

### 2.3. Exposure Definition

Stimulant therapy was defined as the initiation of any of the following medications: methylphenidate (short-acting or extended-release), dexmethylphenidate, or amphetamine-based compounds. The index date was defined as the date of the first stimulant prescription.

### 2.4. Data Analysis

BMI was calculated from documented height and weight measurements in the EMR and converted to age- and sex-specific percentiles using the CDC growth charts. Patients were classified into five BMI categories: Underweight (below the 5th percentile), Normal weight (≥5th–<85th percentile), Overweight (≥85th–<95th percentile), Obesity (≥95th–<99th percentile), and Severe Obesity (≥99th percentile).

Changes in BMI classification were assessed by comparing the baseline (the latest BMI measurement within 12 months before or up to 3 months after treatment initiation) and post-intervention (the latest BMI measurement 6–12 months after treatment initiation) categories.

Descriptive statistics were used to summarize patient characteristics and transitions between body mass index (BMI) categories. Proportions of patients moving to higher or lower BMI categories were calculated for each baseline classification.

### 2.5. Ethical Considerations

The study was approved by the Clalit Health Services Institutional Review Board (Clalit Community Helsinky Committee, protocol number: 0127-23-COM2, Approval date: 2 February 2024). All data were de-identified to protect patient confidentiality.

## 3. Results

### 3.1. Patient Population

Of the 142,916 children in Clalit’s records who were dispensed stimulant medications during the study period and met the criterion of sustained use (≥90 days), only 26,930 (18.8%) met the full inclusion criteria—defined as receiving stimulant treatment for at least 90 days and having BMI data available both before and after treatment initiation. Patient characteristics are presented in Table 1.

When stratified by sex, transition patterns across BMI categories were highly similar between males and females across all baseline BMI groups and age strata (Supplement Table S1).

### 3.2. Baseline BMI Category Distribution

At baseline, 14,238 of children (52.9%) were classified as normal weight, 4163 (15.5%) were classified as overweight (BMI percentile ≥85 <95), 8285 (30.8%) were classified as obese (≥95th percentile), of them 4428 (16.4%) with severe obesity, and 0.9% were underweight.

### 3.3. BMI Category Transitions

Transitions between BMI categories with ADHD medication are presented in Table 2. 

Children with normal weight at baseline: The vast majority (90%) maintained their BMI classification. Only a minority transitioned to overweight (6.5%), obesity (2.3%), and a small number (225, 1.6%), yet important, transitioned to the underweight category.

Children with overweight at baseline: 36% remained overweight, while 48% transitioned to a lower BMI category into normal weight. 16.5% had an increase in BMI category to obesity.

Children with obesity at baseline: 44% remained in the obesity category, 42% moved to a lower BMI category (of them 15% to normal weight), and 510 (13%) transitioned to severe obesity.

Children with severe obesity: 71% remained in their BMI category. 19% transitioned to obesity, 5.5% transitioned to overweight, and 3.7% became normal weight. One patient became underweight.

Among children who were underweight at baseline, 39% transitioned to normal weight, while 61% remained underweight. Interestingly, 1 patient (0.4%) transitioned to the overweight category.

### 3.4. BMI Categories Transition by Age

Transition in weight categories by age with ADHD medication are presented in Table 3. 

Transition to a lower BMI category was more prevalent in the young age group, with 54% of children transitioning from overweight to normal weight and 52% transitioning from obese I to lower BMI categories. Thirty-two percent of young children with severe obesity moved to a lower BMI category. We found no effect of sex on transition in BMI categories.

## 4. Discussion

In this large-scale, real-world cohort study of pediatric patients initiating stimulant therapy for ADHD, we found that stimulant medications are associated with meaningful shifts in BMI classification. Approximately 30% of patients experienced changes in their BMI category during treatment, while 70% remained in their original weight classification.

Children with normal weight were generally weight-stable with ADHD medication treatment, 90% of them remained within the normal BMI %ile range, with only 8.8% transitioning to overweight or obesity. Among the underweight group, 39% improved to a normal weight classification, suggesting some benefit on appetite regulation, while most remained underweight. Children in the higher BMI categories at baseline were least likely to improve their BMI category. While a significant number of children with overweight (48%) reduced their BMI category to normal, in the group of children with severe obesity, only 29% of children transitioned to a lower BMI category. This may indicate a relative resistance to the appetite-suppressing effects of stimulants in this subgroup. Yet, these changes to a lower BMI category are remarkable, even compared to obesity medications. Children with overweight at baseline had the highest likelihood of improvement, with 48% moving to a lower BMI category—although 16% progressed to obesity during the course of treatment.

Younger children seem to be more susceptible to the weight loss effects of ADHD medications, with over 50% of them demonstrating a decrease in weight category following treatment initiation.

This is in agreement with a study that examined weight changes in pediatric patients following initiation of pharmacologic treatments, including stimulants, and found that the most pronounced weight changes were observed in the younger children, with stimulant use associated with weight reduction, while antidepressants and antipsychotics were linked to weight gain [9]. Gurka et al. also analyzed BMI trajectories in children and adolescents receiving ADHD medications, with attention to medication type, age, and sex [10]. Their findings showed an initial decrease in BMI with stimulant use, especially among younger children, though some regained weight or progressed to higher BMI categories over time. This effect in young children is important, as treatment was also found to lead to linear growth reduction in a subgroup of young ADHD patients (younger and shorter at the beginning of treatment), suggesting that an early start of treatment may have long-term growth outcomes [11]. Interestingly, we recently demonstrated that younger children, and children with ADHD treated with medications, adhere better to anti-obesity medications compared to children with ADHD not treated for ADHD [12]. This highlights the need for close monitoring of both height and weight trajectories in young children with ADHD on medication, with heightened attention required when anti-obesity treatments are administered simultaneously.

Our study population included more males; male preponderance is in line with previous research, which showed that the ratio of males to females diagnosed with ADHD in childhood is as high as 2:1 to 10:1 [13,14]. The fact that we found no sex-related effect of treatment on change in weight categories adds important data toward addressing the existing gaps in our knowledge regarding sex-based differences on all levels of ADHD disease manifestation, diagnosis, and therapy [15].

Our findings suggest that although stimulant medications are thought to lead to decreased appetite and BMI %ile reduction, their overall impact on BMI category is variable and relates to age and baseline weight status, and therefore mandates careful monitoring.

The effects of stimulant medications on appetite and metabolism may differ according to baseline BMI category. Children with overweight may experience more pronounced appetite suppression and reduced caloric intake, whereas children with obesity may exhibit compensatory eating behaviors or metabolic adaptations that attenuate weight loss.

## 5. Study Limitations

The primary limitation of the study is that less than 20% of children receiving ADHD medications had pre- and post-BMI measurements. This may lead to a selection bias. However, as our main group of interest was to study the effect of ADHD medications on children with overweight and obesity, we estimate that the study population represents well this group of interest.

A second limitation is that Medication exposure was defined based on dispensing data, and actual adherence could not be directly verified. However, requiring a minimum of three monthly prescriptions was intended to reduce misclassification of short-term use.

Our findings underscore a differential response to stimulant therapy based on baseline weight status and age. Although stimulant medications are known to suppress appetite, their effect on BMI classification varies across age and weight categories. Older children with higher BMI appear to have a greater risk of persisting or worsening obesity despite ADHD treatment, while young children with overweight or normal weight are more likely to maintain or lose weight. This suggests that stimulant-induced appetite suppression may be less effective or more easily compensated for in children with existing obesity, potentially due to behavioral, metabolic, or neurobiological factors.

Clinicians should be aware that while stimulants can promote weight loss in some children, they are not a reliable or consistent strategy for managing obesity. Importantly, a non-negligible proportion of patients, including 16.5% of those with overweight and 13% of those with obesity, experienced further weight gain despite treatment. These data highlight the need for close monitoring of weight trajectories in all children receiving ADHD pharmacotherapy.

Future studies should explore the potential modifying role of cultural and religious context on weight-related outcomes among children treated for ADHD.

Further research is also needed to explore the mechanisms underlying variable weight responses to stimulants and to identify which subgroups may benefit from targeted nutritional or behavioral interventions in conjunction with pharmacological treatment.

Another interesting direction for future research would be to develop a predictive model to identify which children are less likely to lose weight—or may even gain weight—during stimulant therapy to achieve a more personalized treatment regimen.

## 6. Conclusions

Stimulant therapy for ADHD is associated with varying effects on BMI classification in pediatric patients. While a meaningful proportion of children, particularly younger ones who are overweight, experience improvement in their weight status, many children remain within their initial BMI category. Moreover, a notable minority of patients, including those with overweight or obesity, may continue to gain weight during treatment.

These findings highlight the importance of individualized monitoring of weight trajectories in children receiving stimulant medications. Clinicians should not assume uniform weight loss in response to stimulants, especially in children with higher baseline BMI.

## Figures and Tables

**Table 1:** Patient characteristics.

BMI Category Baseline	Overall	Underweight	Normal	Overweight	Obesity	Severe Obesity
n	26,930	244	14,238	4163	3857	4428
%		0.9%	52.9%	15.5%	14.3%	16.4%
Age, Median (IQR)	10 (7, 13)	15 (14, 16)	10 (8, 13)	9 (7, 13)	10 (8, 13)	9 (7, 12)
**Age group,** **n (%)**						
5–6	2988 (11%)	0 (0%)	1429 (10%)	516 (12%)	399 (10%)	644 (15%)
7–12	15,695 (58%)	6 (2.5%)	7992 (56%)	2456 (59%)	2337 (61%)	2904 (66%)
13–17	8247 (31%)	238 (98%)	4817 (34%)	1191 (29%)	1121 (29%)	880 (20%)
**Sex, n (%)**						
Male	17,011 (63%)	157 (64%)	8979 (63%)	2503 (60%)	2350 (61%)	3022 (68%)
Female	9919 (37%)	87 (36%)	5259 (37%)	1660 (40%)	1507 (39%)	1406 (32%)
BMI start, Median (IQR)	18.8 (16.7, 22.1)	15.9 (15.4, 16.4)	16.9 (16.0, 18.6)	19.2 (17.8, 22.2)	21.6 (19.5, 24.9)	25.0 (22.3, 29.1)
BMI start %ile, Median (IQR)	82 (55, 97)	3 (2, 4)	57 (40, 72)	91 (88, 93)	97 (96, 98)	100 (100, 100)

**Table 2:** Transition between BMI categories with ADHD medication.

Baseline BMI	Underweight	Normal	Overweight	Obesity	Severe Obesity
	n = 244	n = 14,238	n = 4163	n = 3857	n = 4428
**End of Follow-Up BMI,** **n (%)**					
Underweight	149 (61%)	225 (1.6%)	0 (0%)	0 (0%)	1 (<0.1%)
Normal	94 (39%)	12,758 (90%)	1985 (48%)	591 (15%)	163 (3.7%)
Overweight	1 (0.4%)	927 (6.5%)	1505 (36%)	1048 (27%)	245 (5.5%)
Obesity	0 (0%)	250 (1.8%)	570 (14%)	1708 (44%)	862 (19%)
Severe obesity	0 (0%)	78 (0.5%)	103 (2.5%)	510 (13%)	3157 (71%)

Background color represents children who did not change their BMI category with ADHD medication.

**Table 3:** Transition in weight categories by age with ADHD medication.

AGE 5–6	BMI Category				
	Underweight	Normal	Overweight	Obesity	Severe Obesity
	n = 0	n = 3276	n = 1157	n = 916	n = 1442
Underweight	0 (NA%)	0 (0%)	0 (0%)	0 (0%)	0 (0%)
Normal	0 (NA%)	2809 (86%)	621 (54%)	232 (25%)	105 (7.3%)
Overweight	0 (NA%)	301 (9.2%)	337 (29%)	247 (27%)	118 (8.2%)
Obesity	0 (NA%)	118 (3.6%)	157 (14%)	297 (32%)	238 (17%)
Severe obesity	0 (NA%)	48 (1.5%)	42 (3.6%)	140 (15%)	981 (68%)
**Age 7–12**	**n = 6**	**n = 6145**	**n = 1815**	**n = 1820**	**n = 2106**
Underweight	2 (33%)	42 (0.7%)	0 (0%)	0 (0%)	0 (0%)
Normal	4 (67%)	5634 (92%)	852 (47%)	251 (14%)	46 (2.2%)
Overweight	0 (0%)	357 (5.8%)	682 (38%)	514 (28%)	100 (4.7%)
Obesity	0 (0%)	94 (1.5%)	246 (14%)	841 (46%)	439 (21%)
Severe obesity	0 (0%)	18 (0.3%)	35 (1.9%)	214 (12%)	1521 (72%)
**Age 13–17**	**n = 238**	**n = 4817**	**n = 1191**	**n = 1121**	**n = 880**
Underweight	147 (62%)	183 (3.8%)	0 (0%)	0 (0%)	1 (0.1%)
Normal	90 (38%)	4315 (90%)	512 (43%)	108 (9.6%)	12 (1.4%)
Overweight	1 (0.4%)	269 (5.6%)	486 (41%)	287 (26%)	27 (3.1%)
Obesity	0 (0%)	38 (0.8%)	167 (14%)	570 (51%)	185 (21%)
Severe obesity	0 (0%)	12 (0.2%)	26 (2.2%)	156 (14%)	655 (74%)

Background color represents children who did not change their BMI category with ADHD medication.

## Data Availability

The data presented in this study are available on request from the corresponding author.

## Supplementary Materials

The following supporting information can be downloaded at: https://www.mdpi.com/article/10.3390/jcm15010044/s1, Table S1: Subgroup analysis by sex.
