# Supplementary material for: The Effect of ADHD Stimulant Treatment on Weight Categories in Children and Adolescents
Source: J Clin Med. 2025 Dec 21;15(1):44. doi: 10.3390/jcm15010044 (PMC12786638; doi:10.3390/jcm15010044)
Supplement: Supplementary file 1 [file jcm-15-00044-s001.zip › jcm-4017356-supplementary.pdf]

Table S1: Subgroup analysis by sex.

Characteristic	Male	Female	Male	Female	Male	Female	Male	Female	Male	Female
	Under weight	Under weight	Normal	Normal	Over weight	Over weight	Obesity	Obesity	Severe obesity	Severe obesity
	N = 157	N = 87	N = 8,979	N = 5,259	N = 2,503	N = 1,660	N = 2,350	N = 1,507	N = 3,022	N = 1,406
**End of follow up BMI, n (%)**										
**Underweight**	95 (61%)	54 (62%)	130 (1.4%)	95 (1.8%)	0 (0%)	0 (0%)	0 (0%)	0 (0%)	0 (0%)	1 (<0.1%)
**Normal**	61 (39%)	33 (38%)	8,029 (89%)	4,729 (90%)	1,260 (50%)	725 (44%)	399 (17%)	192 (13%)	127 (4.2%)	36 (2.6%)
**Overweight**	1 (0.6%)	0 (0%)	587 (6.5%)	340 (6.5%)	808 (32%)	697 (42%)	628 (27%)	420 (28%)	127 (4.2%)	61 (4.3%)
**Obesity**	0 (0%)	0 (0%)	178 (2.0%)	72 (1.4%)	361 (14%)	209 (13%)	1,003 (43%)	705 (47%)	585 (19%)	277 (20%)
**Severe obesity**	0 (0%)	0 (0%)	55 (0.6%)	23 (0.4%)	74 (3.0%)	29 (1.7%)	320 (14%)	190 (13%)	2,126 (70%)	1,031 (73%)
